# Transfected Early Growth Response Gene-1 DNA Enzyme Prevents Stenosis and Occlusion of Autogenous Vein Graft *In Vivo*

**DOI:** 10.1155/2013/310406

**Published:** 2013-03-17

**Authors:** Chengwei Liu, Xuesong Zhang, Shi Wang, Mingxun Cheng, Chuanyu Liu, Shuqing Wang, Xinhua Hu, Qiang Zhang

**Affiliations:** ^1^First Department of General Surgery, The First Affiliated Hospital of Jiamusi University, Jiamusi 154002, China; ^2^Department of Vascular Surgery, The First Hospital of China Medical University, Shenyang 110001, China

## Abstract

The aim of this study was to detect the inhibitory action of the early growth response gene-1 DNA enzyme (EDRz) as a carrying agent by liposomes on vascular smooth muscle cell proliferation and intimal hyperplasia. An autogenous vein graft model was established. EDRz was transfected to the graft vein. The vein graft samples were obtained on each time point after surgery. The expression of the EDRz transfected in the vein graft was detected using a fluorescent microscope. Early growth response gene-1 (Egr-1) mRNA was measured using reverse transcription-PCR and *in situ* hybridization. And the protein expression of Egr-1 was detected by using western blot and immunohistochemistry analyses. EDRz was located at the media of the vein graft from 2 to 24 h, 7 h after grafting. The Egr-1 protein was mainly located in the medial VSMCs, monocytes, and endothelium cells during the early phase of the vein graft. The degree of VSMC proliferation and thickness of intima were obviously relieved compared with the no-gene therapy group. EDRz can reduce Egr-1 expression in autogenous vein grafts, effectively restrain VSMC proliferation and intimal hyperplasia, and prevent vascular stenosis and occlusion after vein graft.

## 1. Introduction

In 1977, Paterson et al. [1] first inhibited gene transcription using a complementary combination of single-stranded DNA and RNA in a cell-free system. Later, Stephenson and Zamecnik [2] reversely inhibited the replication of the Rous sarcoma virus using a 13 oligodeoxynucleotide and pioneered the direction of gene-based drugs by inhibiting gene expression. A variety of catalytic DNA, called DNA enzymes, was one of the important breakthroughs in life science history since the discovery of catalytic RNA (ribozyme, Rz) [3–7].

In 1994, Breaker and Joyce [8] found that a single-stranded DNA molecule (catalytic DNA) can catalyze the hydrolysis of RNA phosphodiester bonds. This single-stranded DNA molecule was also called DNA enzyme (DRz). The enzyme activity center was the “10–23 motif” [9–15] composed of 15 deoxyribonucleotides (5′-GGC TAG CTA CA A CGA-3′). Its mutation or reverse mutation variants had no activities. Both ends of the active center were substrate-binding regions that can specifically combine with the target RNA through the Watson-Crick base pairing.

Early growth response gene-1 (Egr-1) is a Cys2-His2-type zinc-finger transcription factor. A broad range of extracellular stimuli are capable of activating Egr-1, thus mediating growth, proliferation, differentiation, or apoptosis, therefore, participating in the progression of a variety of diseases such as atherosclerosis [16–19]. Previous studies have demonstrated that Egr-1 can activate the restenosis process and intimal hyperplasia and inhibit vascular smooth muscle cell apoptosis in vein grafts [20]. The DNA enzyme is an oligonucleotide that bound to and interfered with translation of the Egr-1 mRNA and it could inhibit the expression of Egr-1. In the present study, an Egr-1 DNA enzyme (EDRz) was designed for Egr-1 mRNA, used a liposome as a carrying agent, and investigated the inhibitory action of the Egr-1 DNA enzyme on vascular smooth muscle cell (VSMC) proliferation and intimal hyperplasia.

## 2. Materials and Methods

### 2.1. Construction of Early Growth Response Gene-1 DNA Enzyme

**Figure 1:**
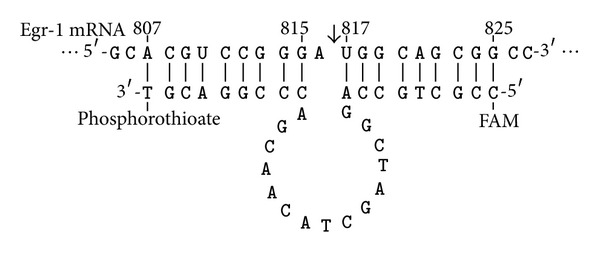
The construction conceptual diagram of Egr-1 DNA enzyme shear and its substrate.

The primer sequences were as follows: 5′-CC GCT GCC AGG CTA GCT ACA ACG ACC CGG ACG T-3′. The 3′ end was phosphorothioate-modified, the 5′ end was labeled with carboxyl fluorescein (FAM), and a total of 15 OD_260_ (495 *μ*g) of the Egr-1 DNA enzyme was synthesized (Figure 1). Approximately 80 *μ*L of DEPC was added to the solution (1 : 1000), mixed, centrifuged, and then added with 120 *μ*L of the liposome Lipofectamine 2000 (Invitrogen,USA). After 10 min, 32 *μ*L of 1 mmol/L MgCl_2_ and 568 *μ*L of Pluronic gel 30% F-127 (Sigma, USA) were added to a final volume of 800 *μ*L. The solution was oscillated and homogenized at 4°C and stored until use.

### 2.2. Establishment of Animal Model and Sample Collection

**Figure 2:**
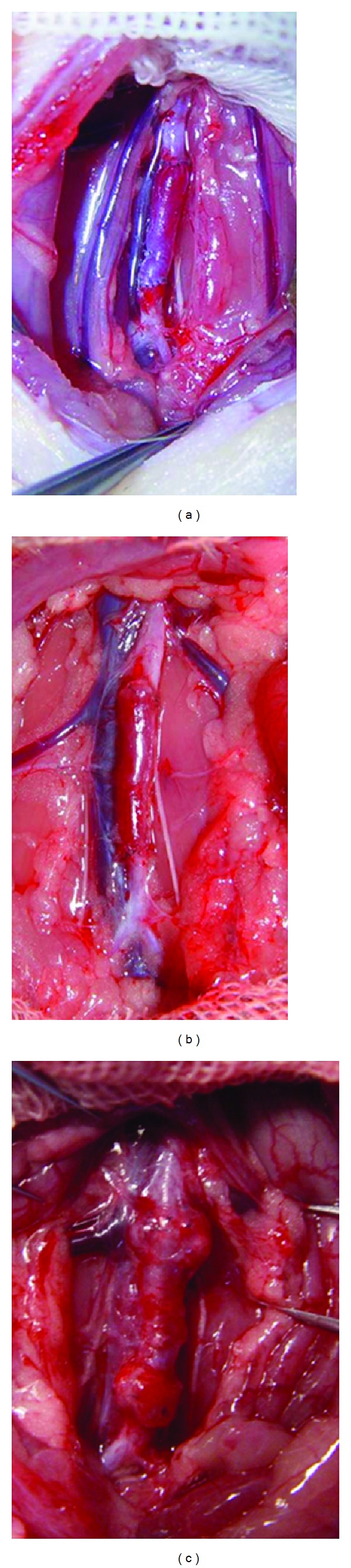
(a) The picture of animal model after graft vein: (b) 28 d after graft in transfection group, the picture of animal model and (c) 28 d after graft in control group, the picture of animal model.

This study was carried out in strict accordance with the recommendations in the Guide for the Care and Use of Laboratory Animals of the National Institutes of Health. The animal use protocol has been reviewed and approved by the Institutional Animal Care and Use Committee (IACUC) of the First Hospital Affiliated to Jiamusi University. Ninety Wistar rats of either sex (200 g to 250 g) were used. The rats were anesthetized with an intraperitoneal injection of 10% chloral hydrate solution (300 mg/kg) and underwent a sterile microsurgery under the SXP-1B microscope (10 times). The procedure was as follows: about 5 mm of the rat's right jugular vein was cut; the vein was flushed with heparin saline; and the vein was anastomosed to the infrarenal abdominal aorta using an 11-0 vascular suture line in an end-to-end manner. Up to 8 *μ*L of EDRz was evenly used around the graft vein (including anastomotic) when no signs of active bleeding were found. The retroperitoneum was closed without the use of anticoagulants either before or after the surgery. The animals were randomly divided into 9 groups (10/group), namely, 1, 2, 6, and 24 h and 3, 7, 14, 28, and 42 days after the graft surgery, respectively. The graft vein specimens were cut on each time point. The no-gene therapy group was taken as control group (Figure 2). 

The specimens were fixed with 4% paraformaldehyde in 0.1% diethylpyrocarbonate (DEPC) for 2 h. The gradient sucrose was dehydrated. Then, they were frozen-embedded and cut into 5 *μ*m thick sections. EDRz transfection on the vein graft was observed under a fluorescence microscope. The localization of EDRz was determined by confocal microscopy. The fluorescence gray value was detected using a fluorescence image analyzer and was replicated and verified in multiple samples.

### 2.3. Histomorphology Staining

Vein grafts were fixed in 10% neutral formalin for 24 h. Conventional dehydration was then performed. The grafts were transparent and wax-dipped. The wax block was embedded and the middle section of the vein graft was cut into 5 *μ*m thick sections. HE staining was conducted and images of the computer image analysis system were collected. Finally, intimal hyperplasia thickness was measured. The data collection and analysis of intimal hyperplasia were performed in a blinded manner.

### 2.4. *In Situ* Hybridization

Specimens were processed with fixation. The sucrose gradient was dehydrated, and frozen-embedded, and then a constant cold slicer was used to slice them into 5 *μ*m thick sections. Digoxigenin-labeled oligonucleotides were used as probes, and the *in situ* hybridization was performed according to the manufacturer's instructions (Wuhan Boster Corporation, Wuhan, China). The specimens were dyed with DAB or AEC. The percentage of positive cells in the total cell in eight-unit perspective was randomly counted and performed in a blinded manner.

### 2.5. Reverse Transcription-Polymerase Chain Reaction

Total RNA was extracted from cell lines according to instructions of the kit (Wuhan Boster Corporation, Wuhan, China). Primers for Egr-1 were designed using the Jellyfish software according to the sequence in GenBank and synthesized by Shanghai Sangon. For Egr-1, the primers were 5′-CAG TCG TAG TGA CCA CCT TAC CA-3′ (Fwd) and 5′-AGG TTG CTG TCA TGT CTG AAA GAC-3′ (Rev), 448-bp long. For *β*-actin, the primers were 5′-TTG TAA CCA ACT GGG ACG ATA-3′ (Fwd) and 5′-GAT CTT GAT CTT CAT GGT GCT-3′ (Rev), 668-bp long. The PCR program involved the following procedures: predenaturation for 2 min at 94°C; denaturation for 30 s at 94°C; annealing for 30 s at 58°C; extension for 1 min at 72°C; and final elongation at 72°C for 10 min. Thirty cycles of PCR were performed. PCR products were analyzed by electrofluorescence on 2% agarose gel in a 1x TAE buffer at a voltage of 100 V for 1 h and EB-stained for 20 min. Band intensity was photographed and analyzed on the Gel Imaging System. The gene expression value = Egr-1mRNA/*β*-actin mRNA.

### 2.6. Immunohistochemical Staining

Conventional SABC staining was performed according to the kit's instructions (Wuhan Boster Company, Wuhan, China). PBS was used in place of primary antibodies as the negative control. The nucleus or cytoplasm had positive brown-yellow (DAB) or red (AEC) particles at 400 times magnification under the light microscope; it was considered positive regardless of dyeing intensity as long as there was a color display. The percentage of positive cells in the total cell in the eight-unit perspective was randomly counted and was performed in a blinded manner.

### 2.7. Western Blot Analysis

The specimens were lysed with a cell lysis solution. The vessel tissues were cut into pieces. The specimens were ultrasound-homogenized. Proteins (100 *μ*g/sample) were separated using 10% SDS-PAGE. The proteins were electrotransferred to nitrocellulose membranes using a semidry system. Then, the membrane was blocked in 5% skimmed milk diluted in TBST for 1 h at room temperature. Thereafter, the membranes were incubated with a primary antibody for 2 h at room temperature. Next, the membranes were further incubated with a horseradish peroxidase-labeled goat anti-mouse IgG antibody at a 1 : 500 dilution. The specimens were washed with TBST three times. Then, 12.5 mg of *β*-Naphthyl acid phosphate and 12.5 mg of O-Dianisidine tetrazotized (Sigma Corporation) were added to color the specimens. The NC membrane was photographed and analyzed on the Gel Imaging System.

### 2.8. Statistical Methods

Data were shown as mean ± SD $\left(\overset{-}{x}\pm s\right)$ and analyzed using the SPSS10 statistical software. The significance of the differences between the group means was determined using ANOVA and post hoc test.

## 3. Results

### 3.1. Egr-1 DNA Enzyme (EDRz) Transfection

**Figure 3:**
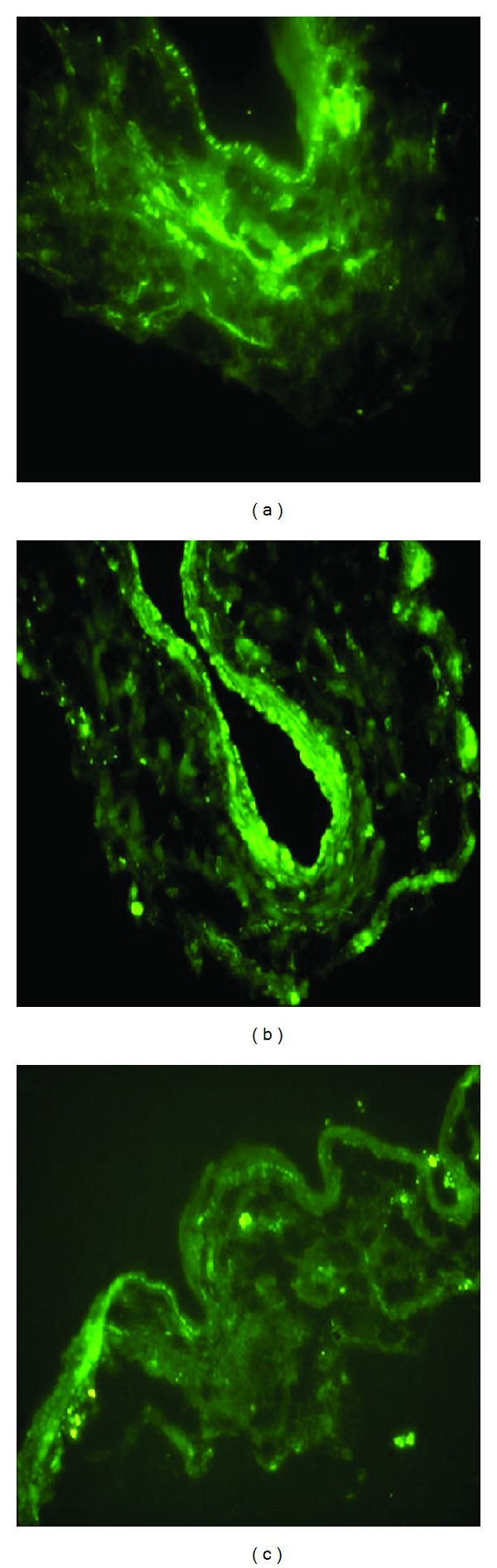
(a) 1 h after graft in transfection group, EDRz located in adventitia, tunica media, and partial endothelial cells by confocal microscopy (×400). (b) 7 d after graft in transfection group, EDRz located in tunica intima by confocal microscopy (×400). (c) 1 h after graft in control group, there was no EDRz in adventitia, tunica media, and endothelial cells by confocal microscopy (×400).

The early growth response gene-1 DNA enzyme was mainly located in the tunica media, adventitia, and partial endothelial cells of the vein graft 1 h after the grafting in transfection group (fluorescence expression value of 70.3 ± 13.5) (Table 1, Figure 3(a)). The early growth response gene-1 DNA enzyme was located in the tunica media of the vein graft from 2 h to 24 h after-grafting. There was a small amount of EDRz in the tunica media of the vein graft 3 d after the grafting. It was mainly located in the intima of the vein graft 7 d after grafting (Table 1, Figure 3(b)). There were no traces of the early growth response gene-1 DNA enzyme in the vein grafts at 14, 28, and 42 d and control group (Table 1, Figure 3(c)).

### 3.2. Changes in Histomorphology

**Figure 4:**
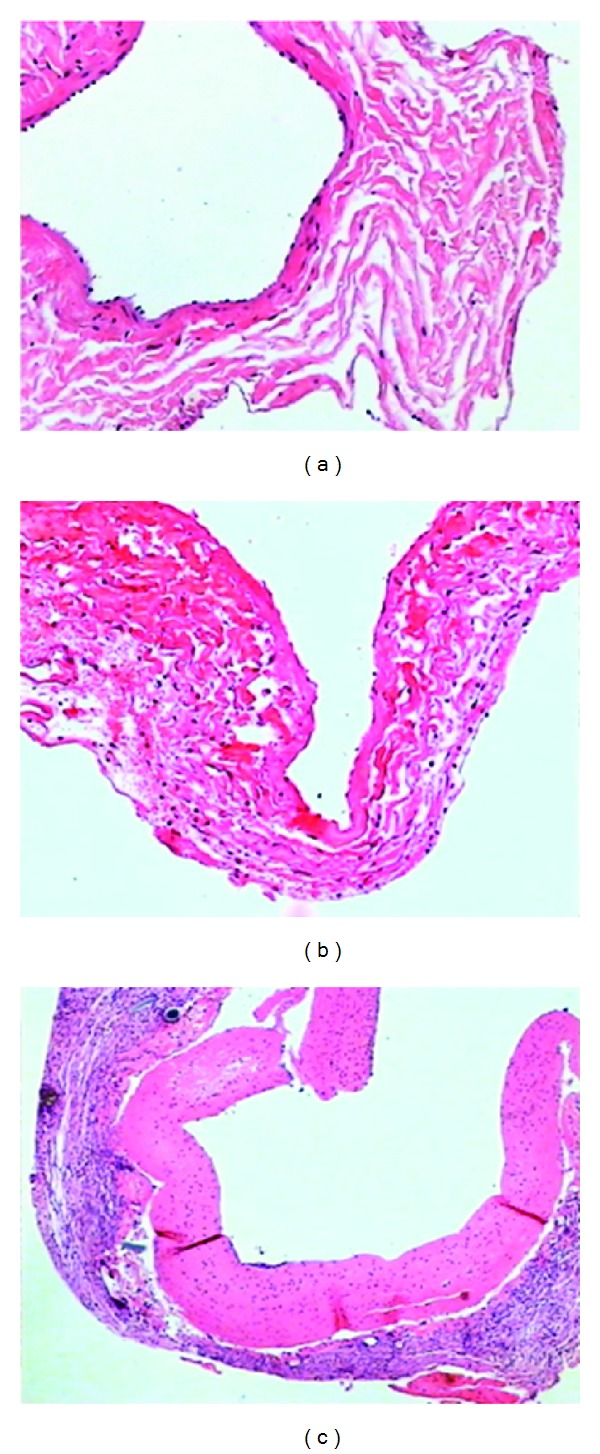
(a) Normal vein (HE × 100). (b) 14 d after graft in transfection group, intimal hyperplasia reached a peak (HE × 100). (c) 14 d after graft in control group, intimal hyperplasia reached a peak (HE × 100).

There was no expression of PCNA protein in normal vein. There was still a small amount of slightly disordered VSMCs in the media 2 h to 6 h after the vein graft compared with the control group. Slightly positive expression of PCNA at 6 h, positive cell rate of (2.5  ±  0.4)% in transfection group, (5.6  ±  0.4)% in control group. Moreover, VSMCs were also found partly in the thin layer of a thrombus formation in the cavity surface of the intima. The intima was partly damaged at 24 h to 3 d after grafting. The expression of PCNA protein was increased from 24 h to 3 d. In addition, endothelial cells were shed and there was a small amount of thrombosis in the local area. The intima thickened and VSMC proliferation was visible at 7 d. Intimal hyperplasia reached a peak (16.4  ±  4.7 *μ*m) at 14 d. The expression of PCNA protein reached peak at 14 d, (15.3  ±  4.2)% in transfection group, (33.5  ±  6.2)% in control group. The vascellum basically completed endothelialization and disordered VSMC were still visible compared with the control group whose degree of VSMC proliferation and thickness of intima were obviously relieved at the same time. The difference was statistically significant (*F* = 3.42, *P* < 0.01). Intimal hyperplasia thickness decreased at 28 and 42 d compared with 14 d and the expression of PCNA protein was decrease (Tables 1 and 2 and Figure 4).

### 3.3. Reverse Transcription-Polymerase Chain Reaction (RT-PCR)

**Figure 5:**
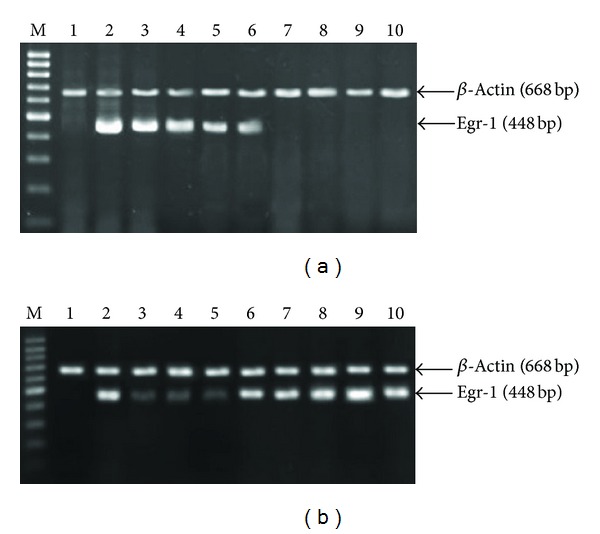
(a) The RT-PCR results of Egr-1 mRNA in transfection group. (b) The RT-PCR results of Egr-1 mRNA in control group. M: Gene Ruler 100 bp DNA Ladder Marker; 1: normal vein; 2–10: transplantation vein at different time after operation, 2: 1 h; 3: 2 h; 4: 6 h; 5: 24 h; 6: 3 d; 7: 7 d; 8: 14 d; 9: 28 d; 10: 42 d.

Egr-1 mRNA expression reached a peak (gene expression value of 1.89  ±  0.63) 1 h after the EDRz was transfected. The expression decreased (0.85  ±  0.42, 0.13  ±  0.03, 0.09  ±  0.04) from 2 h to 24 h after grafting. The expression was weak (0.05  ±  0.01) 3 d after-grafting. There were no more Egr-1 mRNA expressions at 7, 14, 28, and 42 d after grafting (Figure 5(a)). Egr-1 mRNA expression had biphasic changes in control group. Egr-1 mRNA rapid rise at 1 h after graft, a spontaneous decline at 6 h to 3 d, increase at 7 d after graft operation, a peak at 28 days (Figure 5(b)).

### 3.4. *In Situ* Hybridization

**Figure 6:**
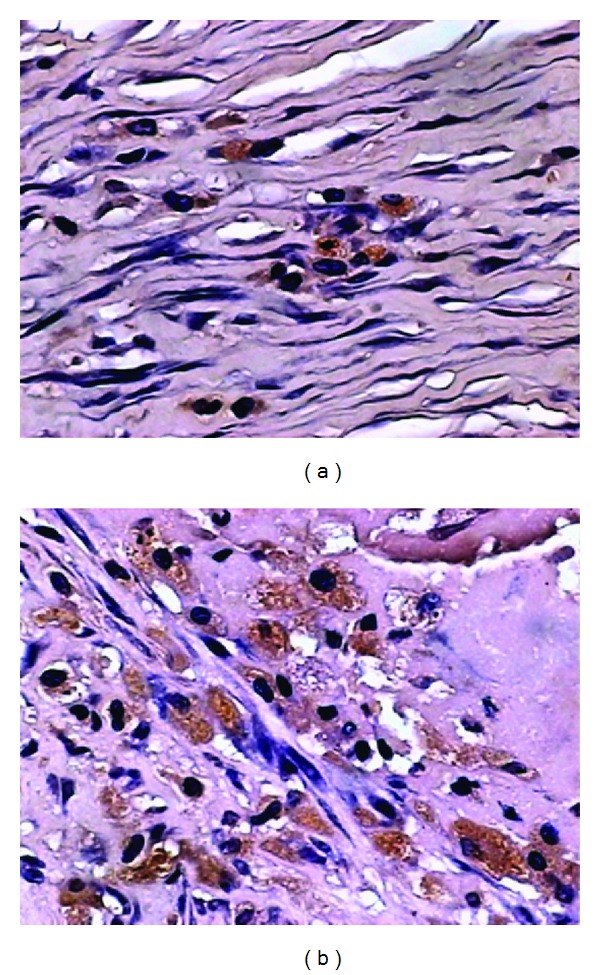
(a) 1 h after graft in transfection group, the positive cells of Egr-1 mRNA were located in cytoplasm of VSMC of neointima by ISH (×400). (b) 1 h after graft in control group, the positive cells of Egr-1 mRNA were located in cytoplasm of VSMC of neointima by ISH (×400).

Partial VSMC showed an Egr-1 mRNA-positive expression in the media of the vein graft 1 h after EDRz transfection. The strongest positive cell expression was (20.1  ±  6.4)%. The difference was statistically significant (*F* = 3.25, *P* < 0.01) compared with the rest of the time points. Its expression decreased from 2 h to 3 d after grafting. There was no Egr-1 mRNA positive expression of neointimal VSMC 7 d after grafting. The trend of positive cells was consistent with the RT-PCR results (Table 3, Figure 6(a)) in control group, and the positive expression of Egr-1 mRNA was found in the part of VSMCs of the media at 1 h after graft. A peak at 28 d, the positive rate of Egr-1 mRNA was (45.7  ±  6.4)%, Egr-1 mRNA major located in the vascular smooth muscle cells of neointimal (Table 3, Figure 6(b)).

### 3.5. Western Blot Analysis

**Figure 7:**
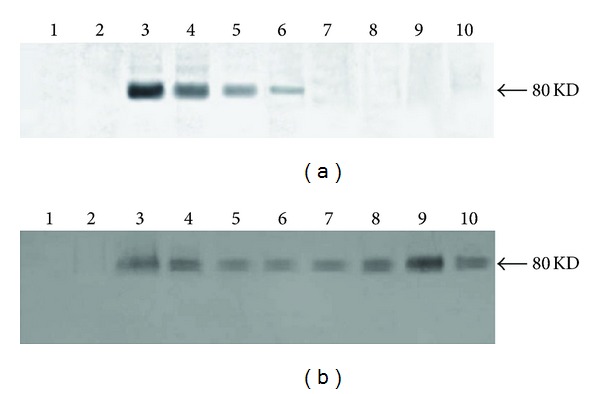
(a) The results of western blot of Egr-1 protein in transfection group. (b) The results of western blot of Egr-1 protein in control group 1: normal vein; 2–10: transplantation vein at different times after operation, 2: 1 h; 3: 2 h; 4: 6 h; 5: 24 h; 6: 3 d; 7: 7 d; 8: 14 d; 9: 28 d; 10: 42 d.

Egr-1-positive cells were not detected in the normal vein. Egr-1 protein expression appeared 2 h after EDRz transfection. The optical density value was (26.4  ±  9.2) × 10^3^. Its expression decreased from 6 h to 3 d after grafting, with optical density values of (14.5  ±  5.2) × 10^3^, (3.4  ±  1.5) × 10^3^, and (2.0  ±  0.8) × 10^3^. Egr-1 positive cells were no longer present 7 d after grafting (Figure 7(a)). In control group, we found that Egr-1 protein was expressed at the early phase of 2 h, and continuing to 6 h, the expression of Egr-1 protein was decline from 24 h to 3 d, reincreased at 7 d, and reached peak at 28 d (Figure 7(b)).

### 3.6. Immunohistochemistry

**Figure 8:**
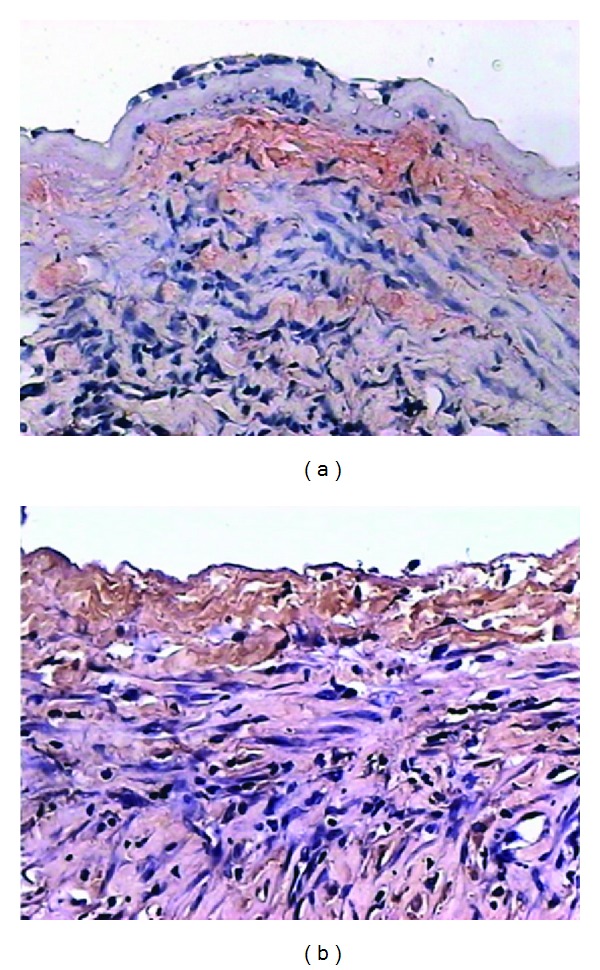
(a) 2 h after graft in transfection group, the positive cells of Egr-1 protein were located in cell nucleus of VSMC of neointima by immunohistochemistry (×200). (b) 2 h after graft in control group, the positive cells of Egr-1 protein were located in cell nucleus of the VSMC of neointima by immunohistochemistry (×200).

The Egr-1 protein was mainly located in the medial VSMCs, monocytes, and endothelium cells during the early phase of the vein graft. However, there were no Egr-1 proteins in the medial and neointimal VSMCs after 7 d. The positive expression rates were as follows: positive cell rate of (15.3  ±  4.2)% at 2h; positive cell rate of (9.7  ±  2.4)% at 6 h; positive cell rate of (6.4  ±  1.8)% at 24 h; and positive cell rate of (2.3  ±  0.2)% at 3d (Figure 8(a)). In control group, the positive expression of Egr-1 protein reached peak at 28 days (40.7  ±  9.5)% (Figure 8(b)).

## 4. Discussion

AUG (816 to 818 sequence) is a selected target of the Egr-1 mRNA. The splice site was located between 816 and 817, adding T GCA GGC CC to the 3′ end of DNA enzyme for the 807–815 sequence (A CGU CCG GG) of Egr-1 mRNA and ACC GTC GCC [21–24] to the 5′ end of DNA enzyme for the 817–825 sequence (UGG CAG CGG). A phosphorothioate modification was made in the 3′ end to resist nuclease degradation, and the 5′ end was labeled with carboxy fluorescein (FAM) for detection purposes. The constructed DNA enzyme was called Egr-1 DNA enzyme (EDRz) (Figure 1). The 816 base (A) of the Egr-1 mRNA did not undergo base pairing with EDRz. Meanwhile, the rest of the EDRz sites formed the combination of base pairing with Egr-1 mRNA. Then, the latter underwent conformational changes. The 2′ end at the point of the OH proton was cut with the help of divalent metal cations, such as Mg2. Moreover, a nucleophilic attack occurred on the adjacent phosphate. The Egr-1mRNA molecular structure was dissociated by two transesterification reactions [25–30].

The substrate-binding site can be applied to shear the RNA of a variety of pathogens and mRNAs of disease-related genes after changing its sequence composition in the 10–23 DNA enzyme [31, 32]. In gene therapy, 10–23 DNA enzymes have the advantages of both the ribozyme (Rz) and antisense oligodeoxynucleotide (ASODN) [33, 34]. The 10–23 DNA enzyme has the following features compared with ASODN: it not only has a substrate RNA antisense inhibitory effect by virtue of the two substrate-binding sites, but also kills virus RNA through the “shear” mechanism [35–38]. Furthermore, DNA enzyme molecules can be used repeatedly, which means that they can shear a number of RNA molecules. The 10–23 DNA enzyme has the following characteristics compared with a variety of Rz: the identified splice site of 10–23 DNA enzyme is present in a range of RNA molecules, including the RNA translation initiation codon AUG of viruses. It is a good shear target and has more shearing targets to choose from compared with Rz. Its nature is relatively stable. The stability of DNA is about 100, 000 times that of RNA in the conditions of physiological pH, temperature, ionic strength, and so on. Its resistance to hydrolysis is about 100 times or more than that of a protein enzyme [39–41]. The sequence of the active center is short. The molecular weight is relatively small with relatively good elasticity. Therefore, it is less affected by the secondary structure of the target sequence. The trend to the substrate is better. Thus, the specificity of the target RNA, combing stability and shear activity, is expressed better than Rz in general [42–44]. It is easier to dissociate the DNA-RNA hybrid molecule than the RNA-RNA hybrid molecule. Therefore, the shear rate of the shear product DRz dissociation process is relatively small [45, 46]. The RNA of the DNA-RNA hybrid molecules can be degraded by the RNA enzyme H. Hence, the DNA enzyme can not only directly kill the target RNA such as Rz, but also cause the hydrolysis of the RNA enzyme H to target RNAs, such as ASODN [47, 48].

The results of this experiment combined with those of previous studies [8, 49, 50] indicated that the early growth response gene-1 DNA enzyme was mainly located in the media and adventitia of the vein graft 1 h after grafting and then gradually shifted to the media. There was a small amount of EDRz in the media of the vein graft 3 d after grafting and was mainly located in the media. It was mainly located in the intima of the vein graft 7 d after grafting. In addition, the Egr-1 DNA enzyme can also be found in some small newborn blood vessels. However, Egr-1 mRNA and protein expressions in the vein graft were not detected 14 d after grafting. There was no EDRz in the vein grafts, suggesting that the EDRz pathway is adventitia → medial → intima and perhaps degraded by a deoxyribonuclease in the end. Egr-1 mRNA and protein expressions decreased at the same time point. Egr-1 mRNA expression decreased obviously 1 h after grafting. This finding indicated that the Egr-1 DNA enzyme rapidly transferred from the adventitia to the media to combine with the Egr-1 mRNA under a short period of time. Hence, the role of the carrier liposome Lipofectamine 2000 was confirmed. Egr-1 proteins were mainly located in the medial VSMCs, monocytes, and endothelium cells during the early phase of the vein graft. However, there were no Egr-1 proteins in medial and neointimal VSMCs 7 d after grafting, indicating that the early growth response gene-1 DNA enzyme can reduce Egr-1 expression in an autogenous vein graft. VSMC proliferation and intimal hyperplasia reached a peak 7 and 14 d after grafting. The degree of VSMC proliferation and thickness of intima were obviously relieved at the same time compared with the no-gene therapy group. Therefore, Egr-1 DNA enzyme transfection of vein grafts with the liposome Lipofectamine 2000 as a carrier can effectively restrain VSMC proliferation and intimal hyperplasia and prevent vascular stenosis and occlusion after vein grafting.

## Figures and Tables

**Table 1:** The result of thickness of intimal hyperplasia in vein graft and EDRz transfection after transfection and no-transfection EDRz ($\overset{-}{x}\pm s$).

	Thickness of intima (*μ*m) in the control group	Thickness of intima (*μ*m) in transfection group	Values of gray scale
Normal vein	2.3 ± 05	2.3 ± 0.5	0
1 h	2.3 ± 0.8	2.3 ± 0.2	70.3 ± 13.5*
2 h	2.3 ± 0.4	2.2 ± 0.3	34.8 ± 9.6
6 h	2.2 ± 0.6	2.3 ± 0.5	25.5 ± 8.7
24 h	2.5 ± 0.3	2.4 ± 0.7	10.1 ± 4.2
3 d	3.2 ± 1.1	3.4 ± 1.2	3.4 ± 1.7
7 d	15.5 ± 2.5	8.9 ± 2.3^#^	1.6 ± 0.8
14 d	26.4 ± 4.7	16.4 ± 4.7^∗#^	0
28 d	30.2 ± 2.8	14.2 ± 3.5^#^	0
42 d	22.7 ± 1.9	9.6 ± 2.8^#^	0

*Compared with other times *P* < 0.01, ^#^compared with control groups *P* < 0.01.

**Table 2:** Contrast of  PCNA protein by immunohistochemistry ($\overset{-}{x}\;\pm \;s$, %).

	Control group	Transfection group
Normal vein	0	0
1 h	0	0
2 h	0	0
6 h	5.6 ± 0.4	2.5 ± 0.4
24 h	9.6 ± 1.5	6.2 ± 1.5
3 d	16.7 ± 2.1	8.3 ± 1.6^#^
7 d	24.6 ± 5.3	10.8 ± 5.7^#^
14 d	33.5 ± 6.2*	15.3 ± 4.2^∗#^
28 d	18.7 ± 9.1	8.1 ± 3.9^#^
42 d	9.8 ± 1.7	3.2 ± 0.6^#^

*Compared with other times *P* < 0.01, ^#^compared with control groups *P* < 0.01.

**Table 3:** Contrast of Egr-1 mRNA and protein by *in situ* hybridization and immunohistochemistry ($\overset{-}{x}\pm s$, %).

	mRNA in control group	mRNA in transfection group	Protein in control group	Protein in transfection group
Normal vein	0	0	0	0
1 h	35.4 ± 7.2	20.1 ± 6.4^∗#^	0	0
2 h	17.8 ± 3.1	10.2 ± 2.3^#^	30.2 ± 5.1	15.3 ± 4.2^∗#^
6 h	8.5 ± 2.2	8.6 ± 1.7	29.4 ± 2.3	9.7 ± 2.4^#^
24 h	8.9 ± 1.6	5.1 ± 1.2	7.2 ± 3.1	6.4 ± 1.8
3 d	8.7 ± 2.4	3.2 ± 0.8^#^	7.2 ± 4.5	2.3 ± 0.2^#^
7 d	15.3 ± 4.5	0	10.8 ± 6.3	0
14 d	25.5 ± 3.6	0	21.6 ± 6.2	0
28 d	45.7 ± 6.8	0	40.8 ± 8.9	0
42 d	28.3 ± 8.4	0	24.1 ± 4.6	0

*Compared with other times *P* < 0.01, ^#^compared with control groups *P* < 0.01.
